# Supplementary material for: Rheological, Thermal, Superficial, and Morphological Properties of Thermoplastic Achira Starch Modified with Lactic Acid and Oleic Acid
Source: Molecules. 2019 Dec 4;24(24):4433. doi: 10.3390/molecules24244433 (PMC6943512; doi:10.3390/molecules24244433)
Supplement: Supplementary file 1 [file molecules-24-04433-s001.pdf]

Supporting Information

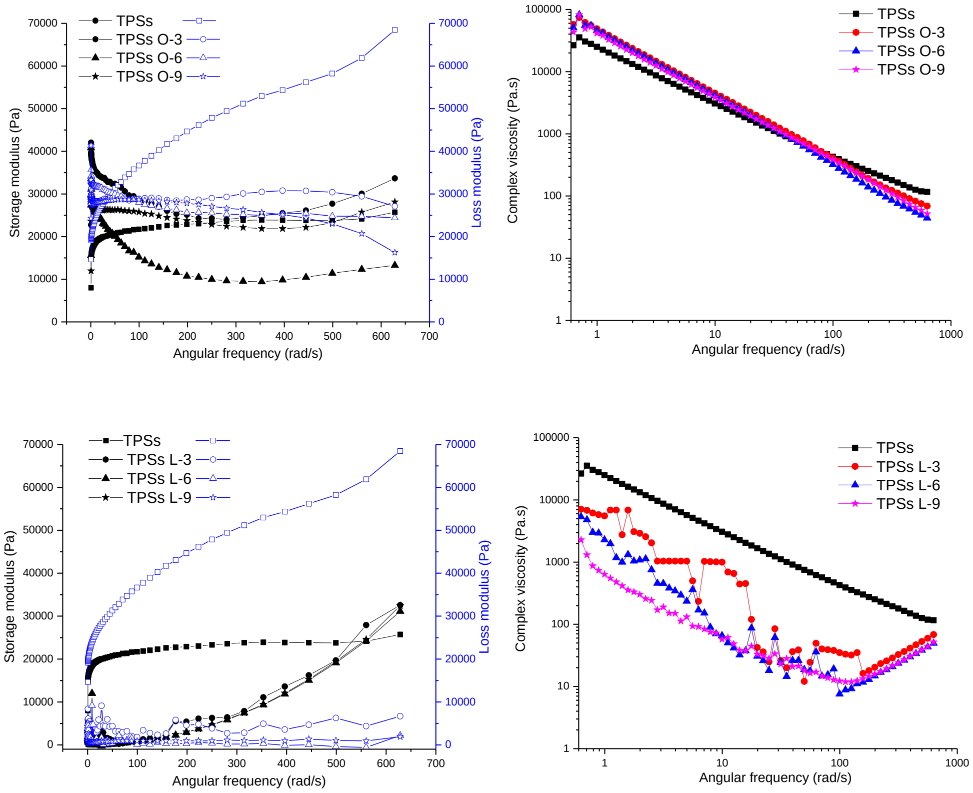

Figure S1. Reograms of TPS samples with sorbitol and different acids. (**a**) Storage modulus (G') and loss modulus (G''), and (**b**) complex viscosity.

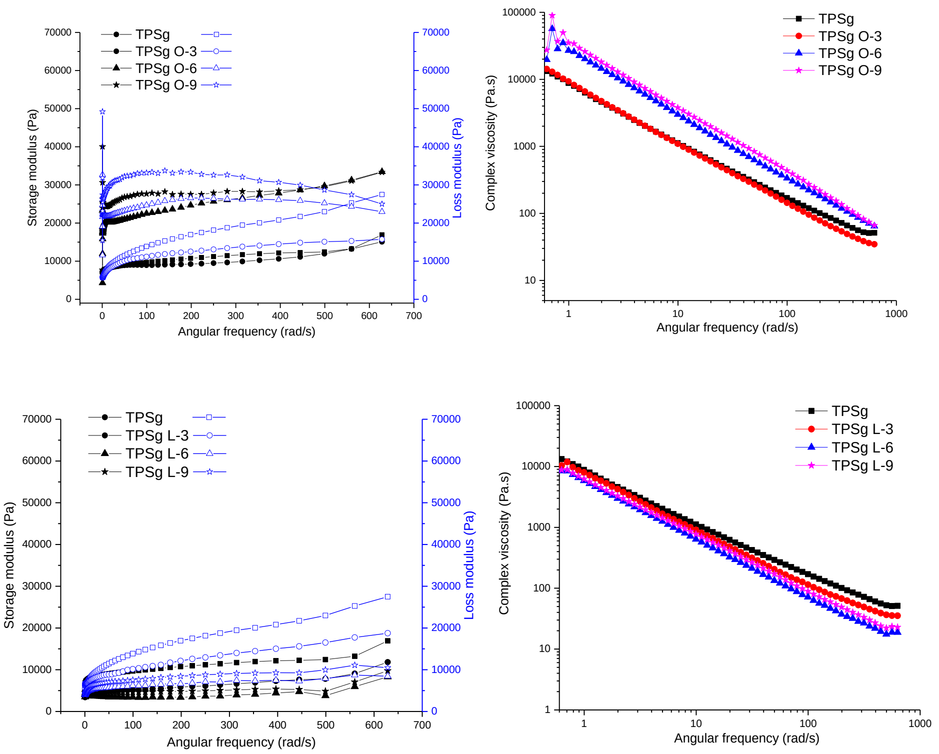

Figure S2. Reograms of TPS samples with glycerol and different acids. (**a**) Storage modulus (G') and loss modulus (G''), and (**b**) complex viscosity.

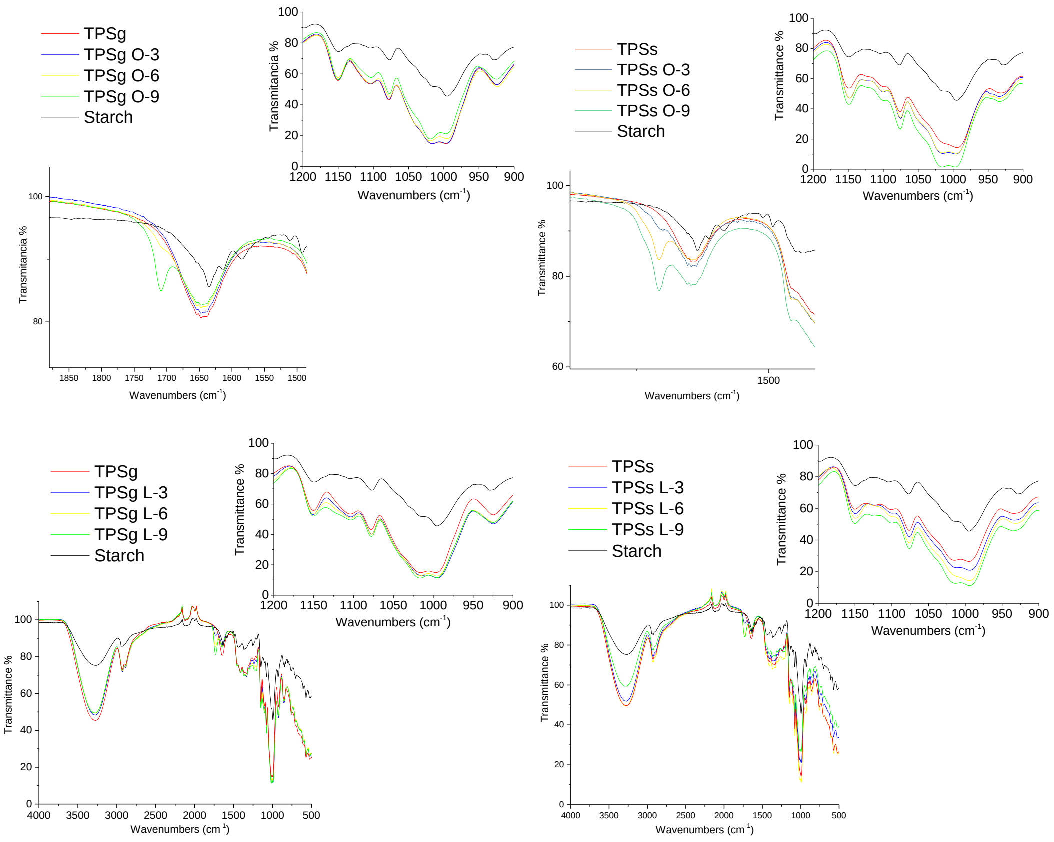

Figure S3. IR spectra of the TPS samples.

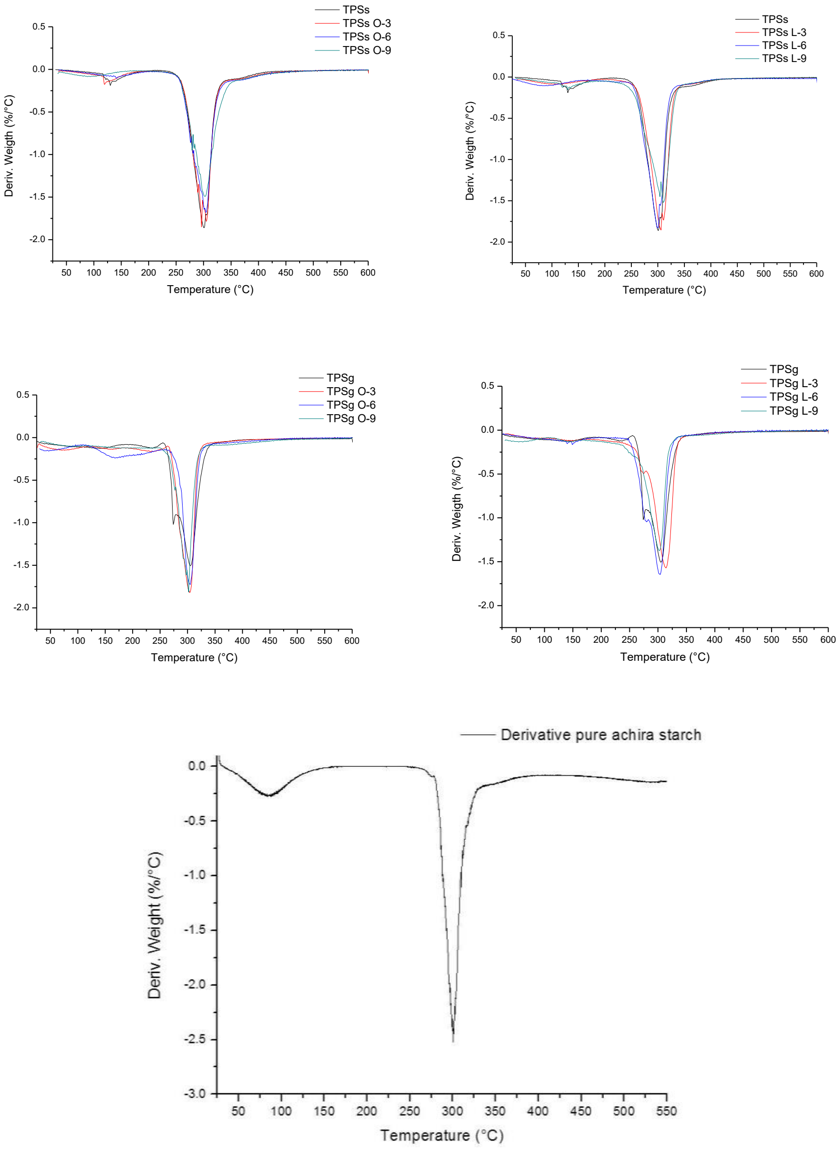

Figure S4. Thermograms with the first weight derivative for TPS samples.
